# Using a 3D virtual muscle model to link gene expression changes during myogenesis to protein spatial location in muscle

**DOI:** 10.1186/1752-0509-2-88

**Published:** 2008-10-22

**Authors:** Ashley J Waardenberg, Antonio Reverter, Christine A Wells, Brian P Dalrymple

**Affiliations:** 1 CSIRO, Food Futures Flagship, Queensland Bioscience Precinct, 306 Carmody Road, St. Lucia, QLD 4067, Australia; 2 Eskitis Institute for Cell and Molecular Therapies, Griffith University, Nathan, QLD 4111, Australia; 3 CSIRO, Livestock Industries, Queensland Bioscience Precinct, 306 Carmody Road, St. Lucia, QLD 4067, Australia

## Abstract

**Background:**

Myogenesis is an ordered process whereby mononucleated muscle precursor cells (myoblasts) fuse into multinucleated myotubes that eventually differentiate into myofibres, involving substantial changes in gene expression and the organisation of structural components of the cells. To gain further insight into the orchestration of these structural changes we have overlaid the spatial organisation of the protein components of a muscle cell with their gene expression changes during differentiation using a new 3D visualisation tool: the *Virtual Muscle 3D (VMus3D)*.

**Results:**

Sets of generic striated muscle costamere, Z-disk and filament proteins were constructed from the literature and protein-interaction databases. Expression profiles of the genes encoding these proteins were obtained from mouse C2C12 cells undergoing myogenesis *in vitro*, as well as a mouse tissue survey dataset. Visualisation of the expression data in VMus3D yielded novel observations with significant relationships between the spatial location and the temporal expression profiles of the structural protein products of these genes. A muscle specificity index was calculated based on muscle expression relative to the median expression in all tissues and, as expected, genes with the highest muscle specificity were also expressed most dynamically during differentiation. Interestingly, most genes encoding costamere as well as some Z-disk proteins appeared to be broadly expressed across most tissues and showed little change in expression during muscle differentiation, in line with the broader cellular role described for some of these proteins.

**Conclusion:**

By studying gene expression patterns from a structural perspective we have demonstrated that not all genes encoding proteins that are part of muscle specific structures are simply up-regulated during muscle cell differentiation. Indeed, a group of genes whose expression program appears to be minimally affected by the differentiation process, code for proteins participating in vital skeletal muscle structures. Expression alone is a poor metric of gene behaviour. Instead, the "connectivity model of muscle development" is proposed as a mechanism for muscle development: whereby the closer to the myofibril core of muscle cells, the greater the gene expression changes during muscle differentiation and the greater the muscle specificity.

## Background

Skeletal muscle exhibits organisation and uniformity in anatomical structure between vertebrate species whilst displaying characteristic differences between muscle types as a functional requirement. This structural organisation is due to the sarcomere, the basic contractile unit and core structural component responsible for the striated appearance of skeletal and cardiac muscle fibres [1,2]. Sarcomeres overlap longitudinally (at the Z-disk) and are joined transversely (at the M-line and the Z-disk) into bundles known as myofibrils. Myofibrils join transversely to the sarcolemma via the costamere, which has been suggested to exist as a skeletal muscle specific extension of the focal adhesions expressed in non-muscle cells and essentially forms the interface between the intracellular and extra-cellular spaces of muscle cells [3-5].

Myogenesis is an ordered process whereby mono-nucleated muscle precursor cells (myoblasts) fuse into multi-nucleated myotubes that mature into the different classes of myofibre [6,7]. Successful skeletal muscle development relies upon the correct cyto-architectural arrangement of the various muscle components and abnormal arrangements can result in devastating effects on muscle function. Indeed, mutations in the genes encoding these components are often associated with muscular diseases including dystrophies [8,9]. One of the major challenges in muscle research is to understand how a muscle precursor cell with a very different organisation develops the highly organised structure responsible for the sophisticated mechanics of muscle. Understanding this fundamental process is of high interest to medical science for treatment of muscle diseases and also animal science for exploring body growth and meat quality determinants. Although muscle development is well-studied, the molecular mechanisms underlying this developmental process remain mostly unknown. Gene expression profiles of myogenesis have primarily focused on the up-regulation of muscle specific genes, for example, genes coding the filamin proteins change from non-muscle-specific to muscle-specific [10-12]. However, restricting analyses to muscle-specific or dynamically changing gene expression sets does not provide an integrated model for understanding the overall function of muscle structural proteins. This is especially true for understanding the transition of broadly expressed to structurally restricted proteins which needs to be underpinned by an understanding of their structure-function relationship.

We have developed the virtual muscle (VMus3D), an animated 3D computer graphics model to visualise the relative locations of the products of muscle gene expression which characterises striated muscle structure at molecular resolution. To test the novelty of the VMus3D in terms of its ability to contribute to the understanding of myogenesis, we have used it to analyse a number of publicly available muscle gene expression datasets.

## Results

### Gene Expression Spatial Visualisation of muscle differentiation using Virtual Muscle and a generic set of costamere, Z-disk and filament proteins

The set of generic mammalian genes encoding the costamere, Z-disk and filament system proteins and their paralogs was built using the published literature and protein-protein interaction databases including BIND (Biomolecular Interaction Network Database; ) and HPRD (Human Protein Reference Database; ). The paralogs were included to enable the analysis and address the issue of paralog swapping. Gene expression data for this set of genes was obtained from a mouse C2C12 *in vitro *muscle cell line before and after differentiation from the NCBI GEO (Gene Expression Omnibus) database [13].

**Figure 1:**
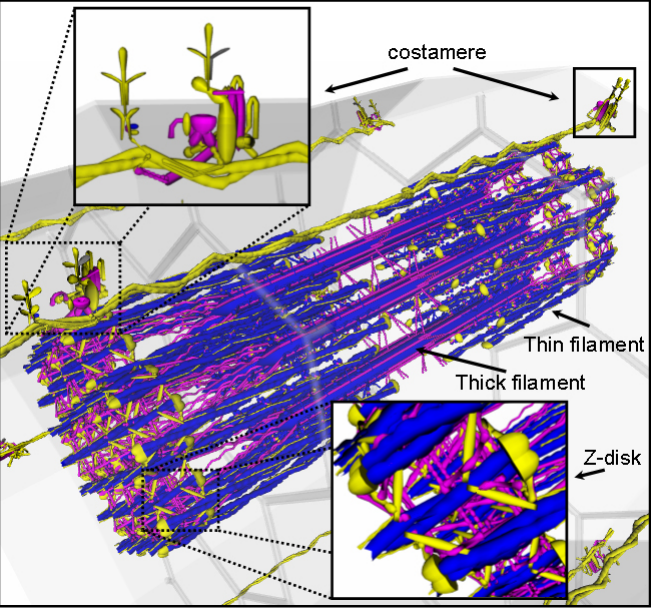
**Spatial gene expression of muscle cell using the VMus3D (myoblast to myotube)**. Initial reconnaissance: The VMus3D demonstrating the spatial gene expression profile of the structure of skeletal muscle from myoblast to myotube. Colours are coded according to gene expression log ratios; yellow: less than two, purple greater than two but less than five and blue greater than five. The filament system demonstrated mostly large changes in gene expression whilst other structural locations did not.

**Figure 2:**
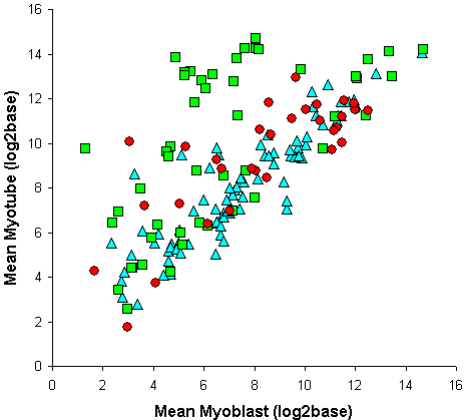
**Gene expression data: myoblast versus myotube (non-filtered)**. Myoblast versus myotube log2base expression means (including paralogs). Costamere: blue triangles; Filament: green squares; Z-disk: red dots.

VMus3D, a database-driven 3D muscle browser of the structural proteins and their arrangements of skeletal muscle (Figure 1), was developed to enable the visualisation of gene expression levels and differences in the relative spatial locations of the proteins encoded by their relevant transcripts. These representative structural arrangements of the VMus3D were compiled from protein-protein interaction data, published experimental localisation data, structural protein data and previously published diagrammatic representations of the contractile apparatus [5,14-19]. Overall differences in the gene expression between myoblasts and myotubes in the mouse C2C12 cell line were shown to have striking concordance with the spatial locations of their protein products (Figure 1). Using VMus3D, the genes that demonstrated the most dynamic up-regulation of gene expression (blue) encode proteins located in the thick and thin filament systems of the myofibril. In contrast, genes encoding proteins located at the costamere and within the Z-disk were expressed less dynamically across the differentiation time-course (yellow/purple) (Figure 1 and 2).

### Gene Expression Profiles of Costamere, Filament system and Z-disk

The limited change in gene expression for the majority of costamere proteins and some Z-disk proteins may have been due to an already increased expression of these genes in the C2C12 myoblasts rather than a genuine consistency of expression level between muscle and undifferentiated cells. To address this question the skeletal muscle specificity (SP_i_: see Materials and Methods: Data Input to the Virtual Muscle) of the expression of each of the genes was ascertained by interrogating the SymAtlas mouse GNF1M dataset which contains a tissue survey of mouse gene expression (see Additional File 1). The expression profiles of the genes encoding the components of the costamere, filament system and Z-disk of muscle, as well as their functionally related paralogs (see Additional File 1) were plotted in three dimensions to include (1) the expression level of myoblasts, (2) the expression level of myotubes and (3) muscle specificity (see Additional File 2). In general, those genes with increased gene expression from myoblast to myotube were also highly muscle specific. The distribution of expression patterns of the genes encoding costamere proteins (and paralogs) remained distinct from those of the Z-disk and the filament systems of muscle. This analysis confirmed that the genes encoding the majority of costamere proteins were not expressed in a muscle-specific manner.

**Figure 3:**
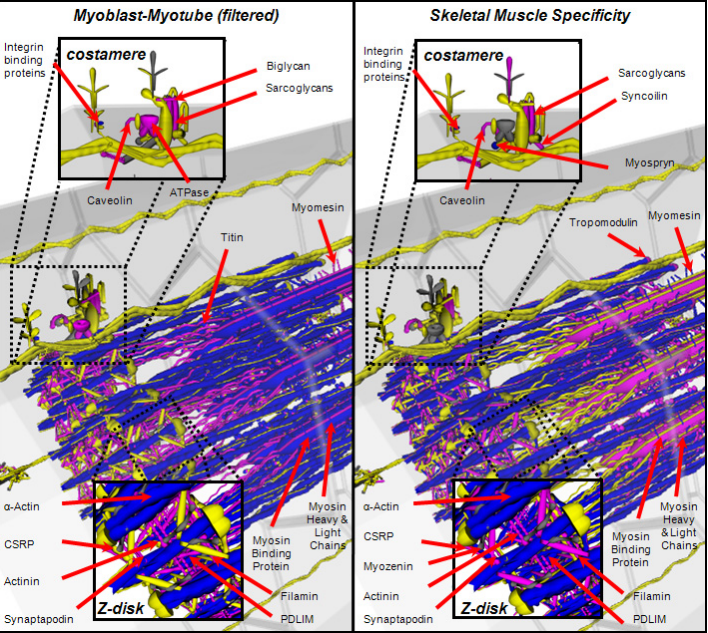
**Spatial gene expression comparison of muscle cell using the VMus3D of myoblast-myotube transition and skeletal muscle specificity index**. Skeletal Muscle Specificity: computed from the difference between the normalised mean expression in skeletal muscle and the median expression in the remaining tissues surveyed. Some differentially expressed components of the myofibril have not been labelled for display simplicity (Troponins and Tropomyosin). Colours are coded according to log ratio thresholds. Yellow: less than or equal to two, purple greater than two but less than equal to five and blue greater than five. Grey indicates no data.

We next superimposed the tissue specificity data onto the VMus3D in comparison to the myoblast to myotube profiles (Figures 1 and 3) to look for additional structural-expression relationships. Again, a clear separation of the costamere gene expression profile from the rest of the myofibril was observed. The proteins of the Z-disc exhibited mixed skeletal muscle specificity. This analysis further revealed a number of genes encoding myofibril proteins that did not show large changes in gene expression across the differentiation time course, and these appeared to be encoded by genes that could be considered muscle-specific (Figure 3). Examples of these genes included Tpm2 in the filament system and Sync of the costamere. These genes may have already been switched on in myoblasts relative to generic cells, poising the myoblasts for differentiation, or be switched on later in muscle development. The relatively high level of expression of these genes in the myoblasts suggests that the first explanation is more likely.

**Figure 4:**
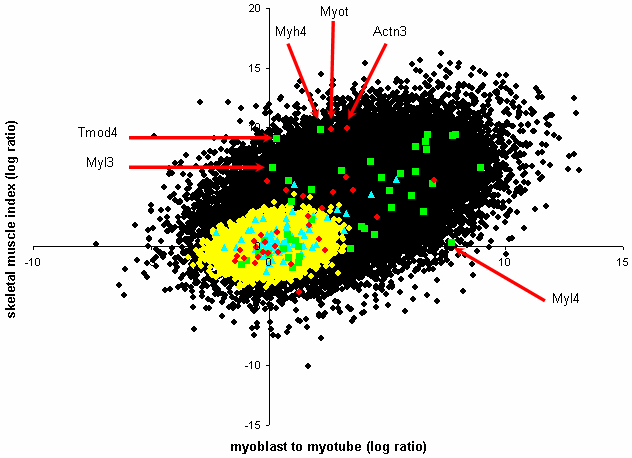
**Clustering of 100,000 simulated components from the two established clusters and structural location within skeletal muscle**. 100,000 components were clustered into the two groups based on the resulting clustering criteria to better establish the boundary between clusters. The genes encoding the proteins belonging to the structural locations, including their paralogs have been grouped. Outliers of different degrees included (1) Tmod4, Myl3 and Myl4 and (2) Myh4, Myot and Actn3. Cluster 1: black dots; Cluster 2: yellow dots; Costamere: blue triangles; Filament: green squares; Z-disk: red dots.

Further examples of developmentally controlled genes were also observed as non-linear outliers when comparing skeletal muscle specificity with myoblast versus myotube gene expression (Figure 4). For example, Myl4 increased expression substantially from myoblast to myotube but had a low adult skeletal muscle specificity index (Figure 4), as it is not expressed in adult skeletal muscle [20]. In addition, Myl3 and Tmod4, which both demonstrated little change in expression from myoblast to myotube, but which had a large skeletal muscle index could be considered to not be expressed in myoblasts or myotubes, possibly representing genes that are switched on later in muscle development (Figure 4). Another non-linear group was also observed that could be regarded intermediate of these observations; containing Myot, Actn3 and Myh4 (Figure 4). Although these genes demonstrated increased expression from myoblast to myotube, judging from their skeletal muscle specificity it can be assumed that they are actually only fully switched on during later muscle development events.

### Two-Component Mixture Model

To further interrogate the observed results, a two-component mixture of bi-variate normal distributions was fitted to the data. The mixture model allowed us to identify the clusters, or components in the mixture, that encapsulated those genes that were simultaneously differentially expressed from myoblasts to myotubes and/or showed skeletal muscle specificity. The two component mixture model analysis successfully defined two distinct clusters (see Additional Files 1 and 3), where clusters 1 and 2 included 57 and 93 genes respectively of the 150 genes analysed. Cluster 1 represented genes that were dynamically expressed from myoblast to myotube and demonstrated muscle specificity whereas cluster 2 did not represent these features. Furthermore, each structural location could then be categorised as having a probability of belonging to each cluster. The filament genes had a 67.5% and 32.5% probability of belonging to cluster 1 and 2 respectively, the costamere genes had a 21.3% and 78.7% probability of belonging to cluster 1 and 2 respectively, while the Z-disk genes had a 46.3% and 53.7% probability of belonging to cluster 1 and 2 respectively.

**Figure 5:**
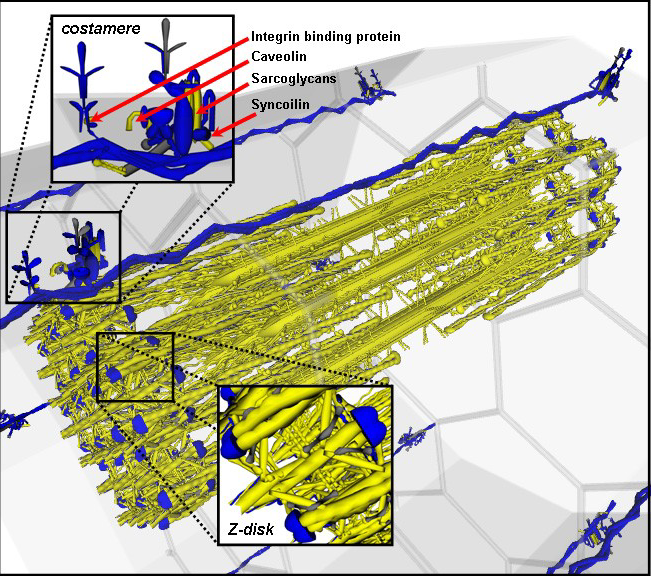
**Using the 100,000 simulated cluster analysis data to visualise two clusters of gene expression as a function of terminal differentiation and tissue specificity**. Blue is belonging to cluster 2 and yellow is belonging to cluster 1. Using the simulated 100,000 component analysis, the original gene expression data was adjusted to these clusters, and assigned to cluster 1 and 2 respectively.

To further enhance the resolution of the boundary defining these two clusters a simulation analysis was performed using 100,000 elements that were generated from a mixture distribution with parameters as estimated from the 150 original data points (Figure 4). From this simulated data, an additional 15 genes that were incomplete for either skeletal muscle specificity or myoblast to myotube data could be assigned a probability of belonging to each cluster and a gene expression value could be estimated (see Additional File 1). Simulation of 100,000 elements and filtering of very low expression values followed by two component analysis permitted the separation of the two observed profiles with greater resolution. Visualising this data using VMus3D provided a clear definition of the clusters relative to their protein spatial locations whilst also establishing that the genes encoding Integrin 1-beta binding proteins 2 and 3 (Itgb1bp2/3), Sarcolgycan alpha and gamma (Scga/g), Caveolin-3 (Cav3) and Syncoilin (Sync) were not conforming to the same cluster as the rest of the components of the costamere (Figure 5). Unlike most costamere genes, these were induced during differentiation and had a high muscle specificity index.

### Paralog exchange during differentiation

**Figure 6:**
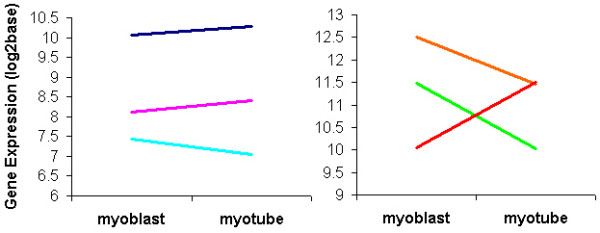
**Example of and lack of paralog exchange**. Left panel: example of Lamb1-1 already being expressed higher relative to other paralogs and its gene expression not changing much from pre- to terminal-differentiation. Lamb1-1:dark blue;Lambc2:pink;Lamb3:light blue. Right panel: example of switching to a tissue specific paralog from myoblast to myotube. Flnc (a component of the Z-disk) which is muscle specific is up-regulated and non-muscle specific paralogs are down-regulated. Flna: orange; Flnb: green; Flnc: red.

Inclusion of paralogs into the structural-based gene expression analysis revealed that down-regulation of a gene often coincided with the up-regulation of expression of another paralog from the same family of genes. Numerous examples of such exchanges were observed in the data and these were often located in the group of genes that were muscle-specific and up-regulated from myoblasts to myotube C2C12 cells. An example is shown in figure 6 (right panel) where non-muscle specific filamins (Flna and Flnb) are down-regulated while muscle-specific Flnc is up-regulated during differentiation. Although this has previously been observed in chickens during myogenesis, the striking concordance of this gene exchange in mouse cells illustrates the similarly of the muscle differentiation process between morphologically distinct vertebrates at this stage of development [21]. Interestingly, paralog exchange was not observed in the group of genes encoding costamere proteins that were not substantially up-regulated during differentiation, a clear example being Lamb1-1 (Figure 6: left panel). This could have been the consequence of earlier paralog exchange (prior to differentiation to myoblasts) if these gene families also display paralog exchanges; or alternative transcripts could be variably expressed in a similar manner but hidden by 'whole gene' expression measurements, that is, measurement of whole gene transcripts irrespective of exon usage.

## Discussion

### Gene Expression Profile and Skeletal Muscle Specificity of Costamere, Filament system and Z-disk during Myogenesis

Clustering of gene expression data followed by grouping on the basis of gene ontology terms or functional annotation is a standard process in the analysis of gene expression data. However this approach tends to neglect those genes that are not differentially expressed and typically categorised as being unimportant, although they may be functionally relevant to the particular biological process under investigation. In our approach we have first grouped genes on the basis of function, in this case on the basis of the complexes which the proteins encoded by the genes are components of, and subsequently analysed changes in gene expression of the muscle system within the context of wider gene expression profiles. Our visualisation of myoblast to myotube differentiation gene expression data using the VMus3D tool resulted in the visualisation of gene expression as a function of time and space during the development of muscle and its relationship to tissue specificity. By observing spatial changes of gene expression during myogenesis, two predominant expression profiles were observed: one profile for which sub-components of the Z-disk and the majority of the costamere did not undergo major changes from myoblast to myotube; and another profile for which the majority of myofibril filament system proteins were substantially up-regulated (Figure 1).

Incorporation of multiple tissue gene expression data into the analysis demonstrated an association of tissue specificity with changes of gene expression from myoblast to myotube. This was supported and refined by a two-component analysis of these genes and their paralogs which demonstrated that two clusters were clearly present as a function of myogenesis and skeletal muscle tissue specificity (Figure 4). By including all available functionally related paralogs for each structural gene in the two-component analysis, this not only improved the statistical power, but also put a biologically relevant level of variance into each of the possible gene states, thus accounting for temporal gene variation whilst enhancing the boundaries of this clustering approach. This inclusion also demonstrated the variability between the different systems and the costamere demonstrated a general lack of this paralog exchange mechanism.

### Connectivity Model of Muscle Development

Many of the costamere proteins have been described as components of the non-muscle specific dystroglycan/sarcoglycan, spectrin and focal adhesion complexes. The Z-disk also appears to form as an early Z-body (initially non-muscle specific) promoting the development of organised sarcomeres, which later integrate muscle-specific proteins to structurally support expanding and contracting myofibres [11,22,23]. Our observations suggest that although the encoded proteins are incorporated into different structures in muscle, the induction of the muscle differentiation process has limited impact on the expression program of many of the genes encoding these proteins. This recruitment of non-muscle specific structures into muscle-specific roles could be coordinated by the presence of the muscle specific components of the costamere which demonstrated up-regulation of gene expression upon differentiation observed herein (Figure 3); Caveolin-3, Syncoilin, Sarcolgycans alpha/gamma and the Beta1 Integrin binding proteins 2 and 3. Each of these components have already been independently recognised as having some involvement in muscle development due to their gene expression profiles [15,24-28], but have not been connected simultaneously to a probable role in the development of the costamere. It is proposed that these genes could represent an initial group of structural proteins bordering intrinsic/extrinsic capability during myotube formation. Evidence has already suggested that the development of pre-myofibril Z-bodies originate from the cell membrane of spreading cardiomyocytes, and that these Z-bodies are aligned to eventually organise into mature Z-discs [23,29]. Therefore, following on in support of previous models, the "connectivity model of muscle development" is proposed: whereby the closer to the myofibril core (i.e. the filament system) of muscle cells, the greater the gene expression changes during muscle differentiation and the greater the muscle specificity index. In addition, the remainder of the genes coding for the costamere proteins that did not demonstrate these activities (i.e. displaying smaller differential and skeletal muscle gene expression changes) leading to the hypothesis that the transcriptome state of the costamere genes in the myoblast may not be different from many other cells in the body.

There are four major mechanisms for the modification of the structures of multi-subunit complexes: (1) recruitment of entirely new proteins; (2) paralog exchange; (3) alternative splice variants; and (4) modification of existing proteins (such as phosphorylation). Construction of the costamere and Z-disk clearly involves the first process, and in some cases the second, although this is most strongly associated with the filament proteins. The relationship of paralog exchange with location also occurs in developed muscle, whereby the filament system also uses paralog exchange as a fundamental mechanism for altering muscle characteristics and hence function; for example via differential incorporation of myosin heavy/light chains and other proteins to drive fibre type changes. In some circumstances, these paralogs maybe co-expressed within an adult cell during fibre type transitions (due to the hybrid nature of myosin heavy/light chains) but the repertoire of paralogs is reduced compared to those expressed and selected for or against during the transition of cell state during early development, as observed herein [30]. In addition, the microarray platform used in these experiments does not enable the resolution of alternative splice variants due to the difficulty in deconvulution of probe sets with regards to individual oligo variances and gene coverage of these oligos in this circumstance being undesirable. Nonetheless, alternative splicing could be contributing to the lack of overall gene expression change if underpinned by changes in splicing patterns during myogenesis. This appears to be a likely explanation for the lack of large differential expression observed for the Alpha and Beta Integrins, which are known to have developmentally regulated alternative splice variants expressed during myogenesis [31]. Finally, gene expression data resulting from microarray hybridizations does not allow exploration of the potential role of protein modification in the structure of multi-subunit complexes.

## Conclusion

In general, the construction of the costamere and to a lesser extent the Z-disk, during muscle differentiation involves the recruitment of broadly expressed proteins. However a small number of genes encoding costamere proteins and more Z-disks proteins are induced during differentiation and these proteins may serve to nucleate the construction of muscle specific structures. Our approach using VMus3D and combining gene expression datasets provides a spatial method of gene expression analysis at molecular resolution in the context of the core structural components of a skeletal muscle cell and these findings highlight the utility of this approach.

## Methods

### Datasets

The mouse C2C12 muscle cell line (a myoblast cell line maintained in growth medium for proliferation that, upon transfer to an appropriate stimulant medium, differentiates into myofibres), and in vitro differentiation have been used in a number of studies [7,13,32]. The gene expression dataset of C2C12 cells differentiated in vitro was retrieved from NCBI GEO [33]; dataset accession number GDS2412 [13]. The user-submitted data (NCBI SOFT format) was used for analysis and the normalisation procedure for the Affymetrix GeneChip Mouse Genome 430 2.0 dataset can be accessed via the accompanying publication [13]. Briefly, the experiment comprised of six microarray hybridisations with two triplicates: one for proliferating myoblasts and another for differentiated myotubes. For each annotated gene and in each of the two conditions (myoblasts and myotubes), the normalised mean expression was calculated from its average log2base transformed expression across the triplicate.

In addition, a mouse GNF1M (MAS5 compressed) dataset was downloaded from the GNF SymAtlas Download portal [34]. This data was combined with the Affymetrix microarray GNF1M annotation file to associate each probe with its corresponding gene symbol. The median expression level of the log2base normalised means of all 61 tissues sampled excluding skeletal muscle and the log2base normalised mean skeletal muscle gene expression level was calculated for each probe.

To eliminate unnecessary relational querying, these two datasets were combined into a single dataset on the basis of matching gene symbols. The criterion chosen (due to multiple probes possibly representing the same gene) was: for every gene symbol from the C2C12 differentiation dataset to include every matching gene symbol and its relative tissue expression level from the processed SymAtlas dataset. Therefore, all possible tissue expression profiles for any of the probes representing a gene were included. Multiple gene expression profiles for a gene were reduced to a single profile for each gene symbol by selecting only the highest normalised mean gene expression value from C2C12 differentiation dataset and tabulated tissue specificity index.

### Construction of the VMus3D

AC3D version 6 was used to build de-convoluted 3D models of the structural proteins and their arrangements within skeletal muscle [35]. The data used for the construction of these simplified structures was restricted to vertebrate evidence due to skeletal muscle structural similarity and included where possible: protein-protein interaction data from public data repositories (HPRD and BIND), published experimental localisation data (e.g. protein localisation), structural protein data (e.g. domain structure: which was also used to determine interaction sites for arrangements of complexes where possible) and previously published diagrammatic representations of the contractile apparatus [5,14-19].

These foundation models formed the basis of an internet accessible browser (Waardenberg et al. in preparation), whereby the models were displayed using X3D (extensible 3D) and web access interaction occurred via its SAI (Scene Authoring Interface) which was supported by Media Machines Flux Player plug-in. Combination of JavaScript, PHP and MySQL databases permitted the querying and compilation of results for real-time injection of data (e.g. gene symbol, expression values and respective colouring) into the X3D scenes, a process known as Ajax (Asynchronous JavaScript and XML) and more recently, Ajax3D when combined with X3D. This injection provided the nodes with the required information to connect with additional internal databases (e.g. to view raw data/different probes/other datasets etc.) and also for connecting to external repositories that relate to the gene expression data chosen.

### Data Input to the Virtual Muscle

The VMus3D, in addition to its accompanying relational database schema also has scripts for transforming and selecting appropriate gene expression values based upon a standard gene set corresponding to the structures within the model for developed adult muscle. For each gene in *i*, two parameters were explored as follows: (1) *MMi*: its differential expression computed from the log ratio of the normalised gene expression values from myoblast to myotube and; (2) *SP*_*i*_: its skeletal muscle specificity computed from the difference between the normalised mean expression in skeletal muscle and the median expression in the remaining tissues (see Additional File 1). Both *MM*_*i *_and *SP*_*i *_were inputs for representation in the VMus3D (Figure 3). The genes included in the VMus3D analysis were also filtered to eliminate genes that were considered to have not been expressed (gene expression levels less than 50, or log2base of ~5.64, in either mean myoblast or myotube) from the C2C12 differentiation dataset. The VMus3D used a colour coded representation of the log ratio thresholds; purple was greater than two, blue was greater than five, yellow was less than two.

### 3D graphing and paralogs

To combine changes in gene expression during C2C12 differentiation relative to muscle specificity, genes were plotted in 3-dimensions (using a X3D graphing application developed for use with the VMus3D): 2 dimensions belonging to time (myoblast and myotube: GDS2412) and another dimension belonging to the difference between the normalised mean expression of a gene in skeletal muscle and its median gene expression across the remaining tissues sampled (GNF SymAtlas). Available paralogs were automatically extracted from the datasets via a script that retrieved all numerical variants of a gene and selected alphabetical subtypes. The retrieved genes and their paralogs were categorised and colour coded into three groups corresponding to the costamere (blue), Z-disk (red) and filament system (green).

### Simultaneous Modelling of Differential Gene Expression and Muscle Specificity

A two-component mixture model analysis was performed on 150 genes (structural genes and their paralogs) to determine if two groups of genes could be classified as distinct clusters based on their differential gene expression from myoblast to myotube and skeletal muscle specificity index observed in the VMus3D (Figure 3). Model-based clustering via mixture of distributions has been proposed by a number of authors to identify differentially expressed genes [36-39]. Similar to the approach of Reverter et al. (2006) for the simultaneous identification of differentially expressed and differentially connected genes, our goal here was to apply model-based cluster analysis to the values of differential expression between myoblast to myotubes (*MM*_*i*_) and muscle specificity (*SP*_*i*_) and for each gene in *i*, and see which genes have relative expression and specificity levels far away from the majority [40]. To this end, and for each gene, the paired data points in *MM*_*i *_and *SP*_*i *_were assumed to be independent observations from a two-component mixture model with probability density function:

$$\begin{array}{c} f\left(\begin{array}{c} MM_{i}\\ SP_{i} \end{array}\right)=\pi_{0}\varphi_{0}\left(\left(\begin{array}{c} MM_{i}\\ SP_{i} \end{array}\right);\mu_{0},V_{0}\right)+\\ \pi_{1}\varphi_{1}\left(\left(\begin{array}{c} MM_{i}\\ SP_{i} \end{array}\right);\mu_{1},V_{1}\right) \end{array}$$

where

$$\varphi_{0}\left(\left(\begin{array}{c} MM_{i}\\ SP_{i} \end{array}\right);\mu_{0},V_{0}\right)$$

denotes the empirical null bi-variate normal density with 2-dimensional mean vector μ_0 _(not necessarily zero) and a 2 × 2 covariance matrix **V**_0 _(not necessarily identity) and corresponding to non-differentially expressed and non-muscle specific genes;

$$\varphi_{1}\left(\left(\begin{array}{c} MM_{i}\\ SP_{i} \end{array}\right);\mu_{1},V_{1}\right)$$

denotes the bi-variate normal density function corresponding to genes that are differentially expressed and/or muscle-specific; finally, the mixing proportions π_0 _and π_1 _are constrained to be non-negative and sum to unity. In the present study, parameters of the mixture model were estimated using the EMMIX-GENE software [36]. Once the parameters were estimated, the posterior probability that the *i*-th gene is differentially expressed and/or muscle specific (i.e., belongs to the second component of the mixture) is given by

$$\tau_{1}\left(\begin{array}{c} MM_{i}\\ SP_{i} \end{array}\right)=\frac{\pi_{1}\varphi_{1}\left(\left(\begin{array}{c} MM_{i}\\ SP_{i} \end{array}\right);\mu_{1},V_{1}\right)}{f\left(\begin{array}{c} MM_{i}\\ SP_{i} \end{array}\right)}.$$

A simulation was also performed by generating 100,000 elements drawn from the estimated two-component mixture model. Genes lacking either skeletal muscle specificity or myogenesis differential expression data were appropriated a probability of belonging to cluster 1 or 2 from their average p-values of the components from the 100,000 simulation that fell into a range of +/-0.1 from the log ratios (see Additional File 1). The VMus3D was then used to display the spatial distribution of the components from the two clusters adjusted using the 100,000 simulation (+/-0.1 myoblast-myotube & +/-0.1 skeletal muscle index) for finer cluster resolution (Figure 4).

## Abbreviations

AC3D: Andy Coulbourne 3D; Ajax: Asynchronous JavaScript and XML; BIND: Biomolecular Interaction Network Database; GEO: Gene Expression Omnibus; GNF1M: Affymetrix Mouse GeneChip Microarray; HPRD: Human Protein Reference Database; SAI: Scene Authoring Interface; Vmus3D: Virtual Muscle 3D; X3D: Extensible 3D.

## Authors' contributions

AJW carried out development and implementation of Vmus3D, normalisation of datasets and analyses of results. AR carried out two-component mixture model analysis. AR, CAW and BPD provided valuable discussion and assistance in manuscript preparation.

## Supplementary Material

Additional file 1 **Costamere, filament and Z-disk genes; including paralogs, gene expression and cluster p-values**. Predicted values highlighted in bold. Click here for file

Additional file 2 **Myoblast, myotube and skeletal muscle specificity plotted in 3-dimensions**. Myoblast versus myoblast log2base expression means including their skeletal muscle specificity index (including paralogs). Costamere: green; Filament: blue; Z-disk: red. Click here for file

Additional file 3 **Cluster analysis of myoblast to myotube including skeletal muscle specificity index**. Initial two clusters calculated. Cluster 1: black dots; Cluster 2; yellow dots. Click here for file
