# Supplementary figures and images for: Using a 3D virtual muscle model to link gene expression changes during myogenesis to protein spatial location in muscle
Source: BMC Syst Biol. 2008 Oct 22;2:88. doi: 10.1186/1752-0509-2-88 (PMC2596796; doi:10.1186/1752-0509-2-88)

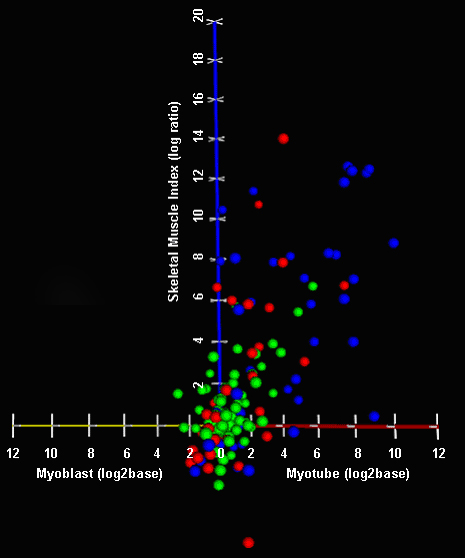

Supplement: Additional file 2 — Myoblast, myotube and skeletal muscle specificity plotted in 3-dimensions. Myoblast versus myoblast log2base expression means including their skeletal muscle specificity index (including paralogs). Costamere: green; Filament: blue; Z-disk: red. [file 1752-0509-2-88-S1.jpeg]

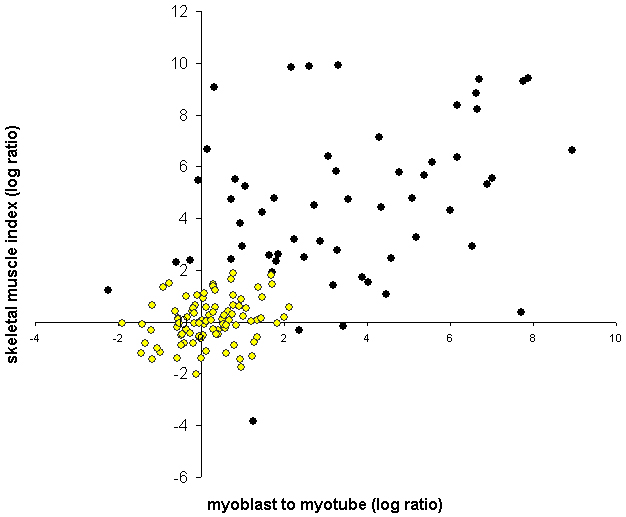

Supplement: Additional file 3 — Cluster analysis of myoblast to myotube including skeletal muscle specificity index. Initial two clusters calculated. Cluster 1: black dots; Cluster 2; yellow dots. [file 1752-0509-2-88-S2.jpeg]
